# Researching Animal Research: What the Humanities and Social Sciences can Contribute to Laboratory Animal Science and Welfare

**DOI:** 10.1017/awf.2024.22

**Published:** 2024-04-19

**Authors:** Erica Fudge

**Affiliations:** University of Strathclyde, Glasgow

The group of scholars and artists involved in this collection titled their Wellcome Trust-funded project the Animal Research Nexus (AnNex). In their introduction, the editors state that ‘nexus’ ‘refers less to an object of study than a way of approaching the complex web of connections that make up animal research’ (p 3). The connections they focus on are teased out through multi-disciplinary approaches from the Humanities and – mainly – Social Sciences. Research undertaken for the project saw more-than-human geographers entering laboratory spaces; historians exploring legislative struggles; artists opening up new ways of engaging publics in ethical discussion. The collection is a culmination of research from the 6-year period of the project (2017–2023) and includes at the end a bibliography of the articles, chapters, theses, and other work produced by the project’s team of scholars. In all, this body of work represents a significant achievement in relation to furthering an understanding of key aspects of animal research and is an excellent advocate for the role of Humanities and Social Sciences scholars in the study and understanding of what has, until now, been the realm of the natural scientists.

The collection follows a clear structure: after the detailed introduction that sets up the project’s aims there are four sections. Each section has a short introduction; a group of three or four stand-alone chapters; and is concluded with short ‘commentaries’ by experts from beyond as well as within the project team. Each of the four sections is focused on a key area: regulation; care; expertise; and openness. The humans involved as participants and as objects of study include policy-makers, citizen scientists, patients, vets, animal technologists, Animal Welfare and Ethical Review Board panellists, and scientists. The essays move between laboratories, breeding centres, fields, theatres, the Houses of Parliament, and universities; and animal research is presented as operating within structures (legal, commercial, educational, social, emotional, ethical) that shape it and which are shaped by it.

It is almost inevitable, given the focus, that questions of visibility and invisibility, of how to bring what is an ethically complex practice into the ‘open’, emerge in different ways across the essays. In her 1998 history of pro-animal activism, *Animal Rights,* Hilda Kean showed the crucial place of making the activities of the closed laboratories visible to the public in nineteenth-century culture; and the question of *seeing* animal research remains central in this collection. In some essays the focus is on outlining and exploring the roles different people play in the activities of animal research and the different pressures they experience. For outsiders, the details offer a valuable insight into what is taking place behind closed doors, literally and metaphorically. But it is not only the outsider who gains insight: the information elicited from the expert interviewees is revealing for those within their fields too. Some of the named veterinary scientists (who were interviewed by Alistair Anderson and Pru Hobson-West) tell stories of how they avoid explaining their work to others, for example, and the animal technologists Sara Peres and Emma Roe spoke to contemplate killing creatures in their care, and the impact that has on them. Patients wonder how much they should know; and how much they want to know about the animal research that lies behind their treatment in discussions with Gail Davies, Richard Gorman and Gabrielle King.

The final section, focused on public engagement activities undertaken by the project members and artists, Bentley Crudgington, Renelle McGlacken, Natalie Scott, Joe Thorne and Amy Fleming, approaches this issue in what Roe calls ‘playful, speculative, and provocative’ ways (p 420), through crafting, performance art, and a creative engagement asking participants to design a label that could be used on medicines to signal that it was produced using animal research. These activities are models for the ways in which arts practices might offer innovative ways of engaging people in complex ethical discussions that are often are closed down by established positions (are you for or against?). They also reveal the value of assuming degrees of emotional and ethical literacy in non-experts that can easily be under-estimated or ignored in public engagement work. As is shown here in the discussion of the ‘Mouse Exchange’, sewing ears on a felt mouse can lead to discussions about care, about individuality and mass; it can produce thoughtful thinking on complex issues.

The issue of who gets included in discussions of animal research comes up on a number of occasions. So, in a chapter in the first section that focuses on legislation and regulation, Dmitriy Myelnikov notes how ‘more radical voices’ (for which read anti-vivisectionists) were excluded in the pursuit of consensus in his history of the origins of the Animals (Scientific Procedures) Act of 1986 (p 30). In a later section, on experts, Alexandra Palmer traces the ways in which citizen scientists (here, amateur ornithologists) are both included in and excluded from scientific work. It is, however, the animals who are excluded from other discussions in the collection. So, Richard Gorman’s essay on horseshoe crabs highlights the vital role their blood plays in the production of vaccines and explores ‘why horseshoe crabs frequently fall outside of current regulations and social imaginations’ (p 59), using this situation to address the complexities of the 3Rs. And Tess Skidmore’s essay on rehoming laboratory animals pays attention to the people involved in making these processes happen, but it doesn’t address the nature of the assessments of the animals, and why some are not deemed fit to be rehomed.

Care is a focus throughout but is addressed specifically in section 2 of the collection. And here the complexities of the issue are brought to the fore. This can be in relation to which species warrant, deserve, or get care: Reuben Message’s study of the aquarists working with zebrafish shows the technologists’ sense of the distance that is felt to be possible from fish, but also notes challenges to that position. There are some fish that can be individualised like ‘Sharkey’, the gigantic zebrafish, an ‘aquarium legend’ who lived to the age of four (p 190). But there is also the experienced contradiction: ‘aquarists dealt with the relative absence of strong inter-species emotional relations in a context that increasingly seems to expect and approve of them’ (p 191). This approval is a focus of Beth Greenough and Emma Roe’s study of the ‘culture of care’ that ‘has become increasingly prominent within animal research’ since 2015 (p 152). Robert GW Kirk recognises the laboratory animal as ‘historically situated’ (p 125), a creature that is ‘a new form of life’ (p 126), and he traces the strain in his study of ‘the moral economy of science’ (p 128): ‘Animal technology discourse adopted technical language whenever possible, yet subjective elements nonetheless supervened because affective and often unsayable experience was recognised to be an essential component of good animal care’ (p 140). As Greenough and Roe note, this abstract conception is experienced at the personal level: the animal technologists ‘as care providers [are] vulnerable to psychological and emotional harm’ (p 166).

In their study of the over-production of animals ‘bred but not used’ Peres and Roe link animal research to the wider commercial cultures in which it operates and write of the technologists’ ‘affective labour,’ and of ‘the emotional and affective resources of those tasked with handling the caring and killing of wasted animal lives’ (pp 293–294). As such, despite their essay’s focus on divisions of labour and outsourcing within laboratory cultures, care is at its core. This can feel strained and, as Eva Giraud notes in her commentary piece, as the model of care that is normalised is one that has its basis in the apparently ‘radically non-anthropocentric’ ideas of contemporary philosophers such as Donna Haraway and Vinciane Despret. Giraud sees a paradox here: instrumental usage is described through the language of engagement and care, which language leaves no space for alternative conceptions of care, such as that voiced by anti-vivisection activists. And it is notable that this paradox is recognised within the laboratories themselves: as one facility manager put it, it is sometimes important to remind recruits to animal technologist positions not to ‘forget why the dogs are here’ (p 166). There is care, but there is also killing, and it is possible to see killing as impacting carers more than animals in the language of the cultures of care.

Overall, this collection offers multiple insights into a key aspect of human relationships with animals in early twenty-first-century culture. It makes clear the value of the Humanities and Social Sciences to our understanding of the work of the activities of the laboratories that are so often hidden from view. But as Louise Mackenzie notes, ‘for the authors … practices of animal research are tacitly accepted and understood’ and this is of course, appropriate: animal research exists and it needs to be understood, to be made available to think with, for and against. However, Mackenzie goes on. This tacit acceptance is assumed in the collection ‘to the extent that the question is not … whether [practices of animal research] should exist at all but rather … how they can exist well’ (p 413). As Giraud’s short contribution makes clear, more challenges to this position might have added further layers to what is undoubtedly an important work.

